# Thin Nevus-Associated Melanoma: Beyond MIA Score Risk Stratification

**DOI:** 10.3390/cancers18152456

**Published:** 2026-07-30

**Authors:** Federico Venturi, Maria Luisa Orlando, Barbara Corti, Paola Valeria Marchese, Francesca Comito, Barbara Melotti, Elisabetta Magnaterra, Biagio Scotti, Aurora Maria Alessandrini, Leonardo Veneziano, Elena Maria Cama, Emi Dika

**Affiliations:** 1 Department of Medical and Surgical Sciences (DIMEC), Alma Mater Studiorum University of Bologna, 40138 Bologna, Italy; 2 Oncologic Dermatology Unit, IRCCS Azienda Ospedaliero-Universitaria di Bologna, 40138 Bologna, Italy; 3 Pathology Unit, IRCCS Azienda Ospedaliero-Universitaria di Bologna, 40138 Bologna, Italy; 4 Division of Medical Oncology, IRCCS Azienda Ospedaliero-Universitaria di Bologna, 40138 Bologna, Italy

**Keywords:** nevus-associated melanoma, thin melanoma, mitotic rate, tumor-infiltrating lymphocytes, risk stratification, early-stage melanoma

## Abstract

**Background**: Nevus-associated melanomas (NAM) are frequently diagnosed at an early stage, yet risk heterogeneity within thin tumors remains poorly characterized. Whether classical histopathologic features can refine risk stratification in this subgroup is unclear. **Objective**: To evaluate the prognostic significance of histopathologic features, particularly mitotic activity and tumor-infiltrating lymphocytes (TILs), in thin nevus-associated melanoma (pTis–pT1a) (<0.8 mm). **Methods**: Retrospective cohort study of 138 consecutive patients with histologically confirmed NAM diagnosed at a tertiary dermatologic oncology center (October 2006–December 2023). Staging was reassessed as per AJCC 8th edition. A predefined subgroup analysis of 100 patients with thin NAM (pTis–pT1a) evaluated adverse outcomes (distant metastasis or death) within 10 years. Associations were assessed using χ^2^ tests and logistic regression. **Results**: Median age was 53.5 years (IQR 44.0–63.8); 60.9% were male. Median Breslow thickness was 0.57 mm (IQR 0.4–0.8). Overall survival was 90.9% at 5 years and 86.0% at 10 years. Among thin NAM, eight patients (8%) experienced adverse outcomes. Mitotic activity ≥ 1/mm^2^ was associated with higher adverse event rates (10.9% vs. 2.8%; OR, 4.30; 95% CI, 0.51–36.43; *p* = 0.18). A gradient was observed across TIL categories: adverse events occurred in 13.8% with absent TILs, 7.3% with non-brisk, and 0% with brisk TILs (*p* = 0.24). Exploratory multivariable analysis showed consistent effect directions without reaching statistical significance. **Conclusions**: Thin nevus-associated melanomas are not uniformly low risk. Mitotic activity and absent TILs were associated with higher adverse event rates, suggesting that classical histopathologic features may refine risk stratification in early-stage NAM.

## Simple Summary

Nevus-associated melanoma, a melanoma arising in association with a pre-existing melanocytic nevus, accounts for approximately one-quarter of all cutaneous melanomas and is usually diagnosed at an early stage. Although thin nevus-associated melanomas generally have an excellent prognosis, a small proportion of patients still develop metastases or die from their disease. Identifying those at higher risk remains challenging. In this study, we investigated whether routinely assessed histopathologic features could improve risk stratification in thin nevus-associated melanoma. We found that increased mitotic activity and the absence of tumor-infiltrating lymphocytes were associated with less favorable outcomes, suggesting that these conventional pathological features may help identify patients who require closer follow-up. These findings provide a basis for future studies aimed at refining prognostic assessment and personalizing the management of early-stage nevus-associated melanoma.

## 1. Introduction

Cutaneous melanoma may arise de novo or in histopathologic association with a pre-existing melanocytic nevus. Approximately 20–30% of melanomas are classified as nevus-associated melanoma (NAM), a proportion that has been consistently reported across large cohort studies and meta-analyses [1,2,3,4].

This distinction is supported by the divergent pathway model of melanoma development, which posits that melanomas arising in the context of high nevus counts and intermittent ultraviolet exposure follow a biologically distinct trajectory from those developing on chronically sun-damaged skin in individuals with low nevus burden [5,6,7,8]. Compared with de novo melanomas, NAMs are consistently associated with younger patient age, truncal location, superficial spreading histologic subtype, lower Breslow thickness (BT), reduced mitotic activity, and less frequent ulceration [2,3,4,9].

Whether these favorable clinicopathologic features translate into an independent survival advantage remains debated. Some studies have reported improved overall survival for NAM in univariable analyses, but this association has generally not been confirmed after adjustment for tumor thickness and stage [9,10]. These findings suggest that the more favorable prognosis observed in NAM may largely reflect earlier detection, potentially facilitated by clinical or dermoscopic surveillance of pre-existing nevi, rather than an intrinsically indolent tumor biology.

Thin melanomas, defined as tumors with a BT of 1.0 mm or less, constitute the majority of newly diagnosed melanomas and carry an excellent overall prognosis, with 10-year survival rates exceeding 95% [11,12,13,14,15,16]. However, thin melanomas are not uniformly low risk: a small but clinically significant subset of patients experience recurrence, metastasis, or death, and approximately 25% of melanoma-related deaths worldwide are attributable to primary T1 tumors [11,12,17,18].

Risk stratification within this subgroup remains challenging. The American Joint Committee on Cancer (AJCC) 8th edition staging system subcategorizes T1 melanomas primarily by Breslow thickness (0.8 mm cutoff) and ulceration status, having removed mitotic rate as a formal staging criterion for T1 tumors [19]. Nevertheless, mitotic rate continues to be recognized as an important prognostic factor across all thickness categories and is recommended for inclusion in pathology reports [20]. Emerging evidence further suggests that additional histopathologic features, including tumor-infiltrating lymphocytes (TILs), lymphovascular invasion, and neurotropism, may refine prognostication in early-stage melanoma beyond current staging parameters [21,22,23,24]. The prognostic role of TILs has attracted particular attention. Population-based and multicenter studies have demonstrated that brisk TIL infiltration is independently associated with improved melanoma-specific mortality and overall survival [22,25,26,27]. In thin melanomas specifically, characterization of the tumor-immune microenvironment has shown promise as a prognostic biomarker, with higher CD8+ T-cell infiltration and favorable CD8+/FOXP3+ ratios associated with reduced recurrence risk [24,28]. However, the prognostic significance of TILs within the specific context of thin NAM has not been systematically evaluated.

Given that NAMs are predominantly diagnosed at an early stage, this subgroup offers a unique opportunity to investigate whether histopathologic features can identify patients at higher risk of adverse outcomes despite apparently favorable staging. To date, most studies comparing NAM and de novo melanoma have focused on broad clinicopathologic differences and overall survival, without dedicated analysis of risk heterogeneity within thin NAMs.

In this study, we present a retrospective cohort of patients with histologically confirmed NAM, with a predefined subgroup analysis of thin tumors (pTis and pT1a), to explore whether mitotic activity, immune infiltrate, and other histopathologic parameters can refine risk stratification within this early-stage population.

## 2. Materials and Methods

We performed a retrospective cohort study, including consecutive patients with histologically confirmed NAM diagnosed at a tertiary dermatologic oncology center between October 2006–December 2023.

All available histopathologic slides and pathology reports were reviewed by experienced dermatopathologists with expertise in melanocytic lesions. For cases diagnosed before adoption of the AJCC 8th edition, the original histopathologic material was re-evaluated to ensure uniform classification across the cohort. NAM was defined as melanoma arising in direct histopathologic association with a pre-existing nevus. Only cases with complete clinicopathologic data and available follow-up information were included. Mucosal and ocular melanomas were excluded.

Previous studies and contemporary clinical practice indicate that NAM are frequently diagnosed at an early stage, often during surveillance of pre-existing nevi. In our cohort, the majority of tumors were classified as pTis or pT1a according to the AJCC 8th edition staging system. Because prognosis in thin melanoma is generally favorable, risk stratification within this subgroup remains challenging. We therefore conducted a predefined subgroup analysis restricted to thin NAM (pTis and pT1a) to explore whether histopathologic features could identify patients at higher risk of adverse outcomes despite being at an early stage at diagnosis.

The following variables were recorded: age at diagnosis; sex; anatomical site; BT; ulceration; mitotic rate (0 vs. ≥1/mm^2^); growth phase (radial vs. vertical); tumor-infiltrating lymphocytes (TILs: absent, non-brisk, brisk); regression (absent vs. present); lymphovascular invasion; microsatellitosis; satellite nodules; AJCC 8th edition pT stage.

For the overall cohort, overall survival (OS) was defined as the time from histologic diagnosis to death from any cause or last follow-up. For the thin subgroup (pTis–pT1a), the primary endpoint was adverse outcome within 10 years, defined as the occurrence of distant metastasis or death within 10 years from diagnosis. This composite endpoint was selected to enhance clinical interpretability and statistical power in a low-event setting.

An exploratory analysis according to the Melanoma Institute Australia (MIA) score was additionally performed within the thin NAM subgroup to evaluate the distribution of adverse outcomes across established sentinel lymph node biopsy (SLNB) risk categories. The MIA risk calculator was developed using data from 3477 patients and uses 6 clinicopathologic input parameters, 3 mandatory (age, BT, and melanoma subtype) and 3 optional (tumor mitotic rate, ulceration, and lymphovascular invasion), to estimate an individual patient’s probability of sentinel node positivity [29,30]. Although the MIA score is not routinely incorporated into Italian AIOM melanoma guidelines, it is occasionally considered during multidisciplinary tumor board discussions in selected borderline cases, in line with NCCN recommendations regarding SLNB risk estimation [31]. For exploratory purposes, patients were stratified according to established MIA score categories (<5%, 5–10%, and >10%), reflecting current thresholds for SLNB recommendation or consideration.

Statistical Analysis

Continuous variables were summarized as median and interquartile range (IQR), and categorical variables as counts and percentages. In the thin subgroup, event rates were calculated across histopathologic features. Associations between categorical variables and adverse outcome were evaluated using χ^2^ tests. Univariable logistic regression was used to estimate odds ratios (ORs) and 95% confidence intervals (CIs). Given the limited number of adverse events, multivariable analyses were exploratory and interpreted cautiously. Statistical significance was defined as a two-sided *p* < 0.05.

## 3. Results

A total of 138 patients with histologically confirmed NAM were included. Median age at diagnosis was 53.5 years (IQR 44.0–63.8), and 60.9% were male. The trunk was the most common anatomical site. Median BT was 0.57 mm (IQR 0.4–0.8), and 75.4% of tumors measured ≤0.8 mm. According to AJCC 8th edition staging, 100 tumors (72.5%) were classified as pTis or pT1a. During follow-up, 22 deaths occurred in the overall cohort. Estimated overall survival was 90.9% at 5 years and 86.0% at 10 years (Table 1).

### Subsection Thin Nevus-Associated Melanoma (pTis–pT1a)

Among the 100 patients with thin NAM, 8 experienced an adverse outcome within 10 years (8%) (Table 2).

Mitotic activity ≥1/mm^2^ was present in 64 patients. Adverse events occurred in 7 of 64 patients with mitotic activity (10.9%), compared with 1 of 36 patients without mitoses (2.8%) (*p* = 0.18) (Table 3).

**Figure 1:**
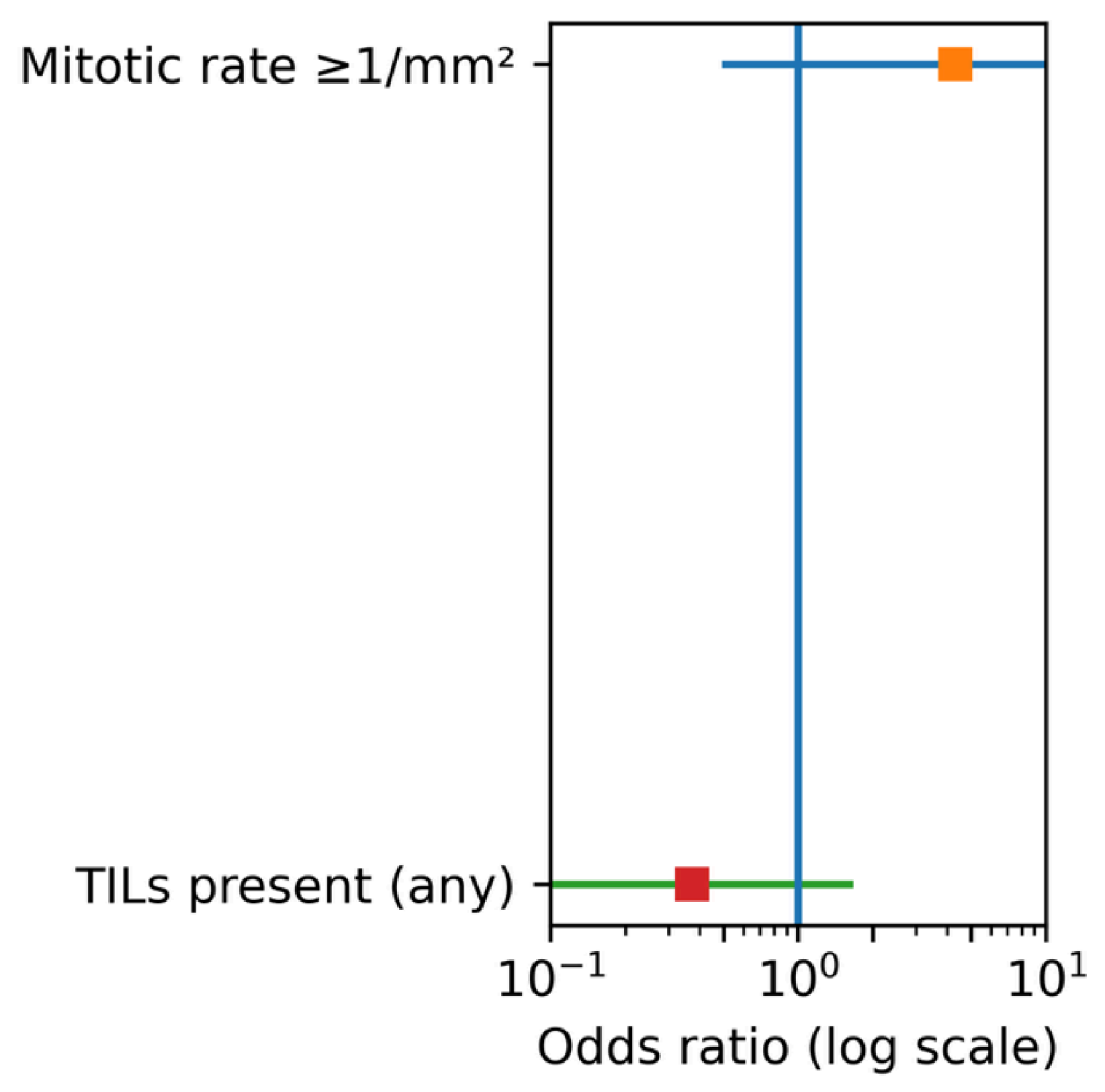
Univariable odds ratios for adverse outcome within 10 years among patients with thin nevus-associated melanoma (pTis–pT1a). Mitotic rate ≥1/mm^2^ was associated with increased risk, whereas the presence of tumor-infiltrating lymphocytes (TILs) appeared protective. Odds ratios are displayed on a logarithmic scale. Adverse outcome was defined as metastasis or death within 10 years from diagnosis. Squares represent odds ratios and horizontal lines indicate 95% confidence intervals.

In univariable logistic regression, mitotic activity ≥ 1/mm^2^ was associated with an OR of 4.30 (95% CI, 0.51–36.43) (Figure 1).

TILs were absent in 29 patients, non-brisk in 55, and brisk in 16. Adverse events occurred in 4 of 29 patients with absent TILs (13.8%), 4 of 55 with non-brisk TILs (7.3%), and none of the 16 patients with brisk TILs (0%) (*p* = 0.24) (Table 2). When TILs were dichotomized (present vs. absent), the presence of TILs was associated with a lower adverse event rate (5.6% vs. 13.8%; OR, 0.37; 95% CI, 0.09–1.61) (Figure 1). Vertical growth phase was uncommon in thin tumors and was not associated with adverse outcome. Ulceration, lymphovascular invasion, microsatellitosis, and satellite nodules were rare in this subgroup and did not show meaningful associations with events. In exploratory multivariable analysis including age, mitotic activity, and TIL presence, no variable reached statistical significance; however, the direction and magnitude of associations remained consistent with univariable findings.

An exploratory analysis was performed to evaluate the distribution of adverse outcomes according to the MIA risk score calculated at diagnosis within the thin NAM subgroup [29,30,32]. Among the 8 patients who developed adverse outcome, 2 were classified within the >10% MIA category and 3 within the 5–10% category, indicating that 5 of 8 patients would have met the current thresholds for SLNB recommendation or consideration. However, adverse outcomes also occurred in three patients classified within the <5% risk category. Although limited by the small number of events, these findings suggest that conventional clinicopathologic prediction tools may not fully capture biologic heterogeneity within thin NAMs. Interestingly, mitotic activity and absence of brisk TILs appeared more consistently associated with adverse outcomes than MIA score category alone (Table 2).

## 4. Discussion

In this dedicated cohort of NAM, the majority of tumors were diagnosed at an early stage and were associated with excellent long-term survival. However, the analysis suggests that thin NAMs (pTis–pT1a) should not be regarded as biologically uniform. Although only 8% of patients with thin NAMs experienced an adverse event within 10 years, risk distribution was not homogeneous. Mitotic activity ≥ 1/mm^2^ was associated with a markedly higher adverse event rate compared with mitosis-negative tumors. While statistical significance was not reached, likely owing to the limited number of events, the magnitude and direction of effect were consistent with established melanoma biology [20]. These findings suggest that mitotic activity may contribute to risk heterogeneity even within early-stage NAMs.

The absence of statistically significant associations for established prognostic variables such as BT and age should be interpreted in the context of the study design. By restricting the analysis to thin melanomas (pTis–pT1a), the variability in BT was intentionally minimized, thereby reducing its discriminatory capacity. Similarly, the relatively homogeneous age distribution decreased the statistical power to detect independent associations for these variables. Therefore, the lack of statistical significance should not be interpreted as evidence that these established prognostic factors are biologically irrelevant, but rather as a consequence of the selected study population and the exploratory nature of the analysis.

Current NCCN guidelines acknowledge that dermal mitotic rate remains an important prognostic factor across all thickness categories and should be included in pathology assessment, despite its removal from formal T1 staging in the AJCC 8th edition [16,33,34]. In thin melanomas specifically, mitotic rate of ≥ 2/mm^2^ has been identified as an adverse feature that increases the risk of sentinel lymph node positivity and may inform decisions regarding sentinel lymph node biopsy [5,35,36,37].

Conversely, a gradient pattern was observed across TILs categories, with decreasing adverse event rates from absent to non-brisk to brisk infiltrates. Notably, no adverse events occurred among patients with brisk TILs. Although underpowered for definitive conclusions, this pattern aligns with growing evidence that host immune response influences melanoma progression from its earliest phases [22,25,26,27]. Within thin NAMs, immune contexture may contribute to differences in biological behavior even among apparently low-risk tumors. Multiple large-scale studies and meta-analyses have demonstrated that brisk TIL infiltration is independently associated with improved melanoma-specific mortality and overall survival [22,25,26]. In thin melanomas specifically, higher CD8+ T-cell infiltration and favorable immune microenvironment profiles have been associated with reduced recurrence risk [23,38]. The absence of adverse events in patients with brisk TILs in this cohort, though based on small numbers, is consistent with these broader findings, and suggests that TIL assessment may have particular value in the risk stratification of early-stage disease [23,26,27,39]. Importantly, the data do not support a distinct prognostic identity of NAM per se. Instead, classical histopathologic features appear to modulate outcomes in a manner consistent with conventional cutaneous melanoma.

The predominance of thin tumors in this cohort likely reflects early detection, potentially facilitated by clinical or dermoscopic surveillance of pre-existing nevi [1,2,3,9]. While NAMs consistently present with more favorable clinicopathologic features (younger age, lower BT, reduced mitotic activity, less ulceration) compared with de novo melanomas, most studies have not demonstrated an independent survival advantage after adjustment for tumor thickness and stage [1,2,9,10]. This suggests that the better prognosis observed in NAMs is largely attributable to earlier detection rather than fundamentally different tumor biology. Nevertheless, this analysis highlights a clinically relevant message: thin NAMs are not uniformly low risk. Histopathologic parameters, particularly mitotic activity and immune infiltrate, may refine risk stratification even at early stages. The 10-year melanoma-specific mortality for T1a melanomas is approximately 1–2%, with overall recurrence rates of 2–8% depending on the presence of adverse features [32,40,41,42,43,44]. Within this generally favorable prognosis group, mitotic rate, TIL status, Breslow thickness >0.4 mm, and other features such as lymphovascular invasion and neurotropism have been identified as predictors of recurrence and metastasis [17,34,37,43,44,45,46,47,48,49]. The current study extends these observations to the specific context of NAM.

The exploratory comparison with the MIA score yielded clinically relevant observations. Current recommendations generally support SLNB for patients with estimated risk >10% and consideration of the procedure for risks between 5% and 10% [29,30]. In our cohort, 5 of the 8 patients who developed adverse outcomes would have met these thresholds based on MIA score estimation at diagnosis, suggesting that the score may retain prognostic utility even within thin NAMs. Nevertheless, adverse outcomes also occurred among patients classified within the <5% category, indicating that a subset of biologically aggressive thin NAMs may not be fully captured by conventional clinicopathologic prediction models alone. Notably, mitotic activity and absence of brisk TILs appeared more consistently associated with adverse outcome than the MIA score category itself, supporting the hypothesis that histopathologic and immune-related features may provide complementary prognostic information beyond established risk calculators.

Several limitations should be acknowledged. The retrospective single-center design and the limited number of adverse events restrict statistical power and preclude definitive prognostic modeling. Accordingly, these findings should be considered hypothesis-generating.

## 5. Conclusions

Thin NAMs (pTis–pT1a) are not uniformly low risk. Although the overall prognosis of this subgroup is excellent, with 92% of patients remaining free of adverse events at 10 years, risk distribution is heterogeneous. Classical histopathologic features appear to modulate outcome in a manner consistent with conventional cutaneous melanoma, suggesting that nevus association does not confer a distinct prognostic identity. Mitotic activity ≥1/mm^2^ was associated with a nearly fourfold increase in adverse event risk compared with mitosis-negative tumors, consistent with established evidence that mitotic rate retains prognostic significance across all melanoma thickness categories [44,50,51].

Conversely, a gradient pattern was observed across TIL categories, with no adverse events occurring among patients with brisk TILs, supporting the hypothesis that immune contexture may represent a marker of biological restraint even in early-stage disease [52]. Future directions include the prospective validation of histopathologic risk stratification in thin nevus-associated melanoma, exploration of quantitative or AI-assisted TIL scoring methods to enhance prognostic accuracy, and investigation of emerging biomarkers such as gene expression profiling or circulating tumor DNA to complement conventional clinicopathologic assessment [53,54,55,56,57,58,59]. Integration of molecular and immune profiling with traditional staging parameters may ultimately enable more personalized risk stratification and inform decisions regarding surveillance intensity, sentinel lymph node biopsy, and potential adjuvant therapy in early-stage melanoma [17,58,60,61,62,63,64,65]. In summary, thin nevus-associated melanomas should not be regarded as biologically uniform. Histopathologic features, particularly mitotic activity and TILs, may contribute to risk stratification within this early-stage population.

The exploratory comparison with MIA score categories further suggests that conventional clinicopathologic prediction models may incompletely capture biologic heterogeneity within thin NAMs, particularly in tumors characterized by mitotic activity and limited immune infiltrate. Population-based validation studies have demonstrated that neither the MIA nor the MSKCC nomogram provides net benefit at risk thresholds ≤5%, with clinical utility demonstrated only at thresholds of ≥8–10% [66,67]. Given that the majority of thin NAM patients fall below the 5% predicted risk threshold, these findings underscore the need for refined risk stratification tools that incorporate immune microenvironment parameters alongside conventional clinicopathologic variables.

## Figures and Tables

**Table 1:** Baseline demographic, clinicopathologic, and follow-up characteristics of the entire cohort of patients with nevus-associated melanoma (*n* = 138).

Characteristic	Value
Age at diagnosis, median (IQR), years	53.5 (44.0–63.8)
Male sex, *n* (%)	84 (60.9)
Anatomic site, *n* (%)	
–Head and neck	21 (15.2)
–Trunk	78 (56.5)
–Upper extremities	18 (13.0)
–Lower extremities	19 (13.8)
–Other sites	2 (1.5)
Breslow thickness, median (IQR), mm	0.575 (0.4–0.8)
Breslow ≤ 0.8 mm, *n* (%)	104 (75.4)
Ulceration present, *n* (%)	9 (6.5)
Mitotic rate ≥ 1/mm^2^, *n* (%)	92 (66.7)
Growth phase vertical, *n* (%)	32 (23.2)
Tumor-infiltrating lymphocytes, *n* (%)	
–Absent	39 (28.3)
–Non-brisk	83 (60.1)
–Brisk	16 (11.6)
Regression present, *n* (%)	54 (39.1)
Lymphovascular invasion, *n* (%)	3 (2.2)
Microsatellitosis, *n* (%)	2 (1.4)
Satellite nodules, *n* (%)	4 (2.9)
AJCC 8th edition pT stage, *n* (%)	
–pTis	3 (2.2)
–pT1a	96 (69.6)
–pT1b	12 (8.7)
–≥pT2	25 (18.1)
Thin tumors (pTis–pT1a), *n* (%)	100 (72.5)
Deaths during follow-up, *n* (%)	22 (15.9)

**Table 2:** Clinicopathologic characteristics of patients with adverse outcomes within the thin nevus-associated melanoma subgroup (pTis–pT1a).

Patient	Age	Sex	Breslow (mm)	Mitotic Activity	TILs	Ulceration	MIA Category
1	55	M	0.50	0/mm^2^	Non-brisk	No	<5%
2	30	F	0.60	>2/mm^2^	Non-brisk	No	>10%
3	63	M	0.45	<2/mm^2^	Absent	No	<5%
4	59	F	0.40	<2/mm^2^	Non-brisk	No	5–10%
5	70	M	0.40	<2/mm^2^	Non-brisk	No	5–10%
6	65	M	0.25	<2/mm^2^	Absent	No	>10%
7	64	F	0.45	<2/mm^2^	Absent	No	5–10%
8	45	F	0.35	<2/mm^2^	Absent	No	<5%

**Table 3:** Histopathologic risk patterns associated with adverse outcome within 10 years in patients with thin nevus-associated melanoma (pTis–pT1a). Adverse outcome was defined as the occurrence of distant metastasis or death within 10 years from diagnosis. *p*-values were calculated using the χ^2^ test. TILs, tumor-infiltrating lymphocytes.

Feature	No. of Patients	Adverse Events ≤ 10 y, *n* (%)	*p*-Value
Mitotic rate			0.18
0/mm^2^	36	1 (2.8)	
≥1/mm^2^	64	7 (10.9)	
Tumor-infiltrating lymphocytes (TILs)			0.24
Absent	29	4 (13.8)	
Non-brisk	55	4 (7.3)	
Brisk	16	0 (0.0)	

## Data Availability

The data that support the findings of this study are available on request from the corresponding author.

## Abbreviations

The following abbreviations are used in this manuscript:

NAM	Nevus-Associated Melanoma
AJCC	American Joint Committee on Cancer
TILs	Tumor-Infiltrating Lymphocytes
IQR	Interquartile Range
ORs	Odds Ratios
CIs	Confidence Intervals
